# The genome sequence of the two-banded wasp hoverfly, *Chrysotoxum bicinctum* (Linnaeus, 1758)

**DOI:** 10.12688/wellcomeopenres.17382.1

**Published:** 2021-11-24

**Authors:** William Hawkes, Karl Wotton, Matt Smith, University of Oxford and Wytham Woods Genome Acquisition Lab, Natural History Museum Genome Acquisition Lab, Darwin Tree of Life Barcoding collective, Wellcome Sanger Institute Tree of Life programme, Wellcome Sanger Institute Scientific Operations: DNA Pipelines collective, Tree of Life Core Informatics collective, Darwin Tree of Life Consortium

**Affiliations:** 1 Centre for Ecology and Conservation, University of Exeter, Penryn, UK; 2 Independent Researcher, Reading, UK

**Keywords:** Chrysotoxum bicinctum, two-banded wasp hoverfly, genome sequence, chromosomal, Diptera

## Abstract

We present a genome assembly from an individual female *Chrysotoxum bicinctum *(the two-banded wasp hoverfly; Arthropoda; Insecta; Diptera; Syriphidae). The genome sequence is 913 megabases in span. The majority of the assembly (98.81%) is scaffolded into five chromosomal pseudomolecules, with the X sex chromosome assembled.

## Species taxonomy

Eukaryota; Metazoa; Ecdysozoa; Arthropoda; Hexapoda; Insecta; Pterygota; Neoptera; Endopterygota; Diptera; Brachycera; Muscomorpha; Syrphoidea; Syrphidae; Syrphinae; Syrphini; Chrysotoxum; *Chrysotoxum bicinctum* (Linnaeus 1758) (NCBI:txid323313).

## Background

*Chrysotoxum bicinctum*, the two-banded wasp hoverfly, is one of Britain’s most distinctive hoverflies. Its chocolate-coloured wing markings and bright yellow bars on the second and fourth abdominal segments make this fly unmistakeable in the field ( Ball *et al*., 2015; van Veen, 2010). The genus *Chrysotoxum* are large wasp-mimic hoverflies with long, elegant antennae and consist of more than 110 species ( Masetti *et al*., 2006). This wasp mimicry likely gives protection against predation by birds through batesian mimicry ( Leavey *et al*., 2021). Across their flight period of May to September, this species is common across southern Britain but its abundance decreases with northerly latitude. *C. bicinctum* inhabits grassy meadows and open woodland rides feeding on a range of flowers but with a preference for composites and umbellifers ( Ball *et al*., 2015). Very little is known about the larval biology of this hoverfly but it is thought that they feed upon the root aphids residing within the nests of *Lasius niger* ants ( Speight, 1976). Observations of ovipositing behaviour include a female *C. bicinctum* repeatedly laying eggs about a *Lasius* ant nest ( Rotheray & Gilbert, 1989). It is not known how the hoverfly larvae avoid predation by the ants who are usually highly protective of their root aphid charges. Potential avenues of research include pheromone mimicry of the aphids by the hoverfly larvae, or simple armour to negate the attacks of the ants. Insight into the biological life history of this distinctive hoverfly is currently severely lacking. It is hoped that with this production, for the first time, of a high quality *Chrysotoxum bicinctum* genome, generated as part of the Darwin Tree of Life project, will further aid understanding of the biology and ecology of this hoverfly.

## Genome sequence report

**Figure 1.**
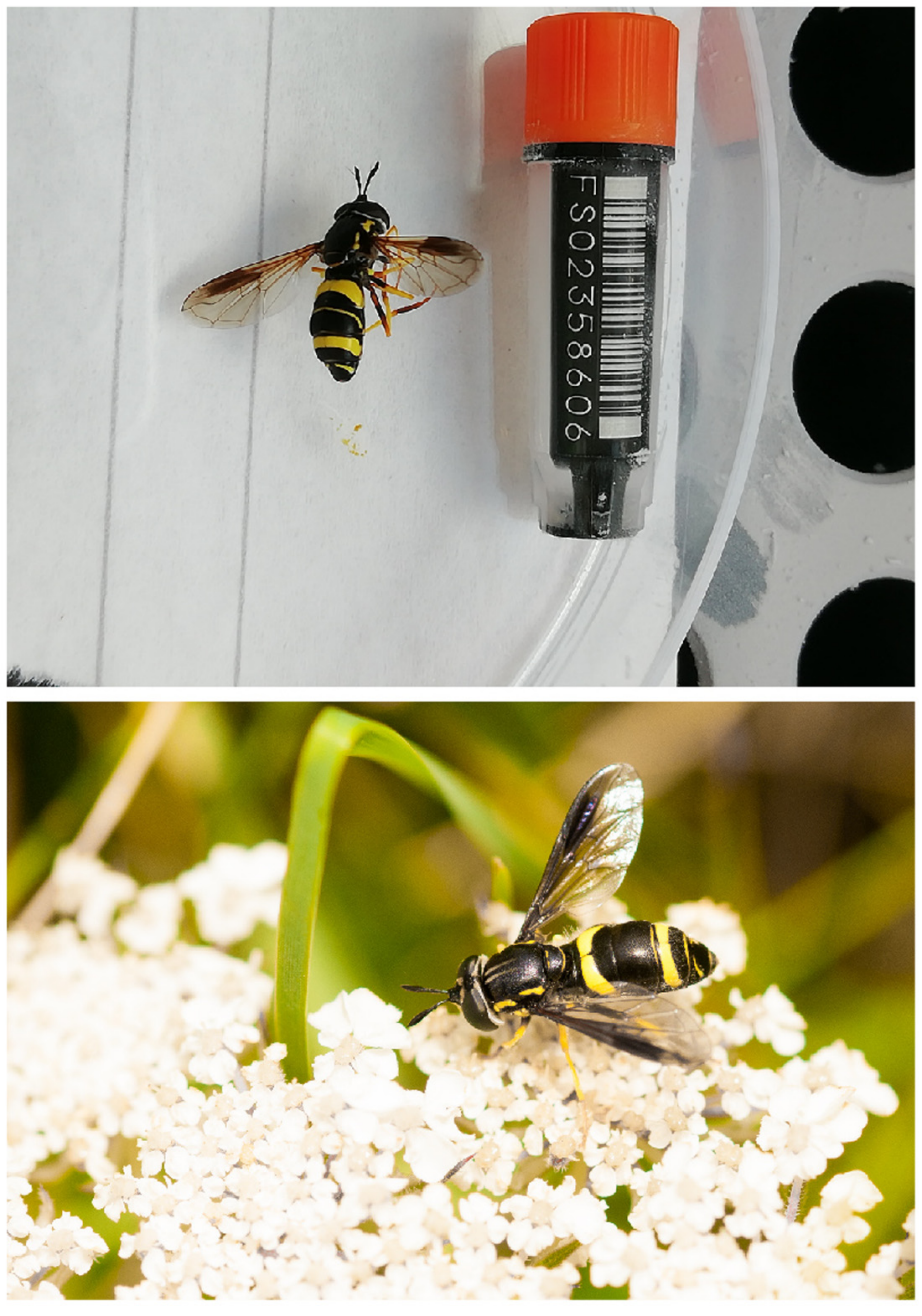
Top, image of the idChrBici1 specimen used for sequencing, with 43.9 mm FluidX sample tube for scale. Below, example image of a *Chrysotoxum bicinctum* individual (not the sampled specimen) taken by William Hawkes.

The genome was sequenced from a single female *C. bicinctum* ( Figure 1) collected from Wytham Great Wood, Oxfordshire, UK (latitude 51.769, longitude -1.33). A total of 29-fold coverage in Pacific Biosciences single-molecule long reads and 37-fold coverage in 10X Genomics read clouds (from molecules with an estimated N50 of 60 kb) were generated. Primary assembly contigs were scaffolded with chromosome conformation Hi-C data. Manual assembly curation corrected 326 missing/misjoins and removed 87 haplotypic duplications, reducing the assembly length by 3.27% and the scaffold number by 64.06%, and increasing the scaffold N50 by 644.27%.

**Figure 2.**
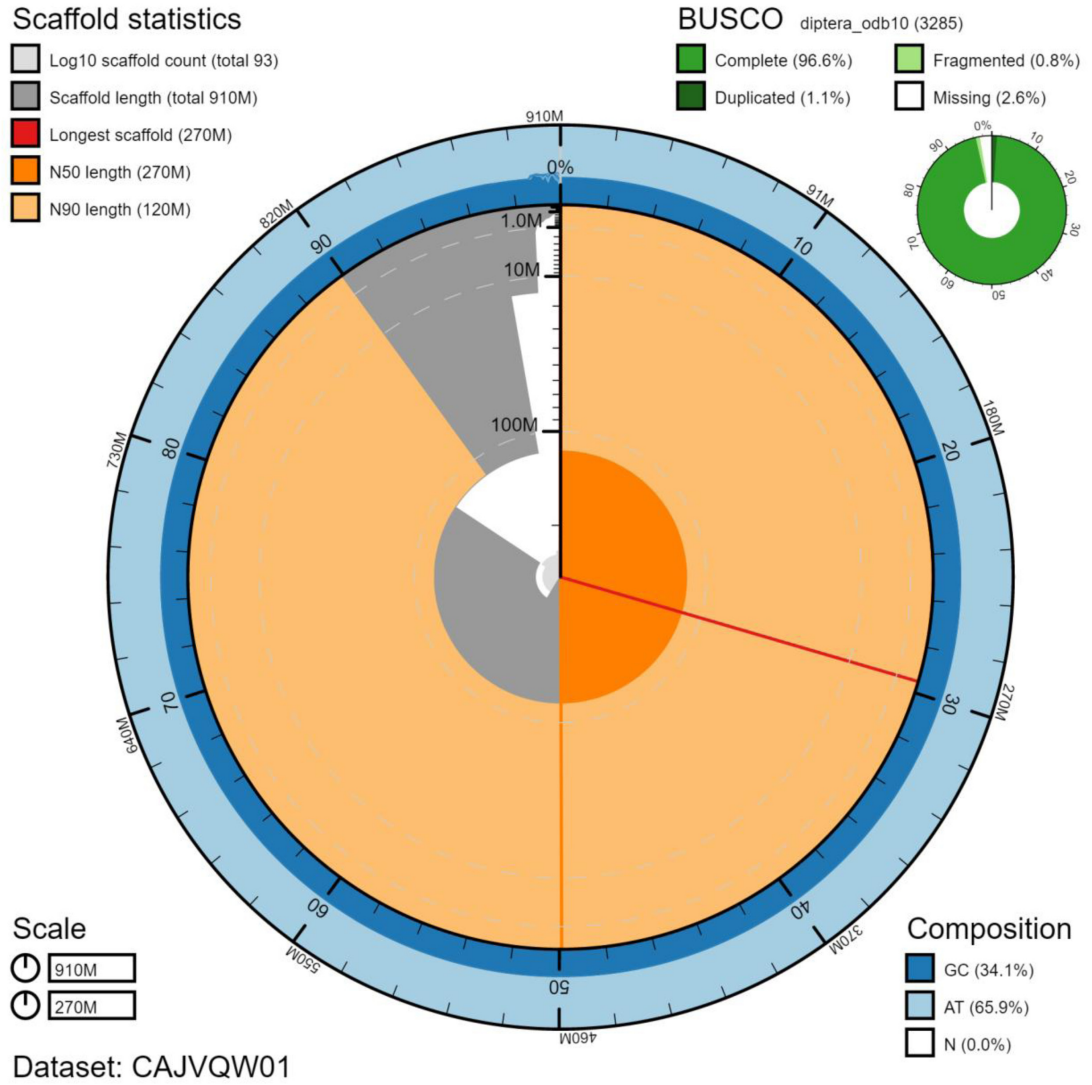
Genome assembly of *Chrysotoxum bicinctum*, idChrBici1.1: metrics. The BlobToolKit Snailplot shows N50 metrics and BUSCO gene completeness. The main plot is divided into 1,000 size-ordered bins around the circumference with each bin representing 0.1% of the 912,938,338 bp assembly. The distribution of chromosome lengths is shown in dark grey with the plot radius scaled to the longest chromosome present in the assembly (269,711,166 bp, shown in red). Orange and pale-orange arcs show the N50 and N90 chromosome lengths (265,788,494 and 117,573,787 bp), respectively. The pale grey spiral shows the cumulative chromosome count on a log scale with white scale lines showing successive orders of magnitude. The blue and pale-blue area around the outside of the plot shows the distribution of GC, AT and N percentages in the same bins as the inner plot. A summary of complete, fragmented, duplicated and missing BUSCO genes in the diptera_odb10 set is shown in the top right. An interactive version of this figure is available at https://blobtoolkit.genomehubs.org/view/idChrBici1.1/dataset/CAJVQW01/snail.

The final assembly has a total length of 913 Mb in 92 sequence scaffolds with a scaffold N50 of 118 Mb ( Table 1). The majority, 98.81%, of the assembly sequence was assigned to 5 chromosomal-level scaffolds, representing 4 autosomes (numbered by sequence length), and the X sex chromosome ( Figure 2– Figure 5; Table 2). The assembly has a BUSCO ( Simão *et al*., 2015) completeness of 96.6% (single 95.5%, duplicated 1.1%) using the diptera_odb10 reference set. While not fully phased, the assembly deposited is of one haplotype. Contigs corresponding to the second haplotype have also been deposited.

**Table 1.** Genome data for *Chrysotoxum bicinctum*, idChrBici1.1.

*Project accession data*	
Assembly identifier	idChrBici1.1
Species	*Chrysotoxum bicinctum*
Specimen	idChrBici1 (genome assembly, Hi-C); idChrBici2 (Hi-C, RNA-Seq)
NCBI taxonomy ID	323313
BioProject	PRJEB45198
BioSample ID	SAMEA7520032
Isolate information	Female, head/thorax, abdomen (idChrBici1); Unknown sex, head/ thorax, abdomen (idChrBici2)
*Raw data accessions*	
PacificBiosciences SEQUEL II	ERR6412376, ERR6558188
10X Genomics Illumina	ERR6054965-ERR6054968
Hi-C Illumina	ERR6054969-ERR6054971
Illumina polyA RNA-Seq	ERR6464930
*Genome assembly*	
Assembly accession	GCA_911387755.1
*Accession of alternate haplotype*	GCA_911387745.1
Span (Mb)	913
Number of contigs	412
Contig N50 length (Mb)	5.7
Number of scaffolds	92
Scaffold N50 length (Mb)	265.8
Longest scaffold (Mb)	269.7
BUSCO * genome score	C:96.6%[S:95.5%,D:1.1%],F:0.8%,M:2.6%,n:3285

*BUSCO scores based on the diptera_odb10 BUSCO set using v5.1.2. C= complete [S= single copy, D=duplicated], F=fragmented, M=missing, n=number of orthologues in comparison. A full set of BUSCO scores is available at https://blobtoolkit.genomehubs.org/view/idChrBici1.1/dataset/CAJVQW01/busco.

**Figure 3.**
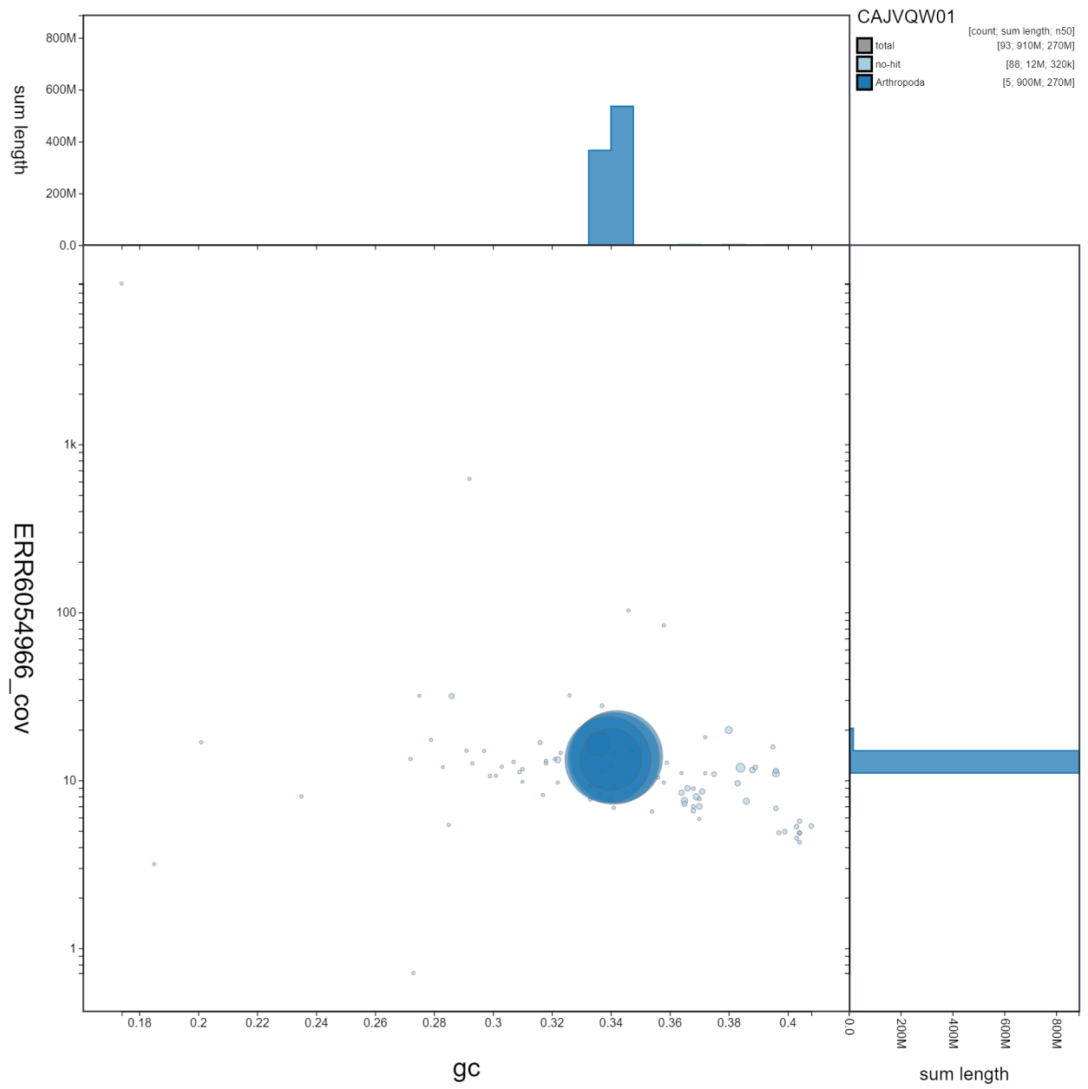
Genome assembly of *Chrysotoxum bicinctum*, idChrBici1.1: GC coverage. BlobToolKit GC-coverage plot. Scaffolds are coloured by phylum. Circles are sized in proportion to scaffold length. Histograms show the distribution of scaffold length sum along each axis. An interactive version of this figure is available at https://blobtoolkit.genomehubs.org/view/idChrBici1.1/dataset/CAJVQW01/blob.

**Figure 4.**
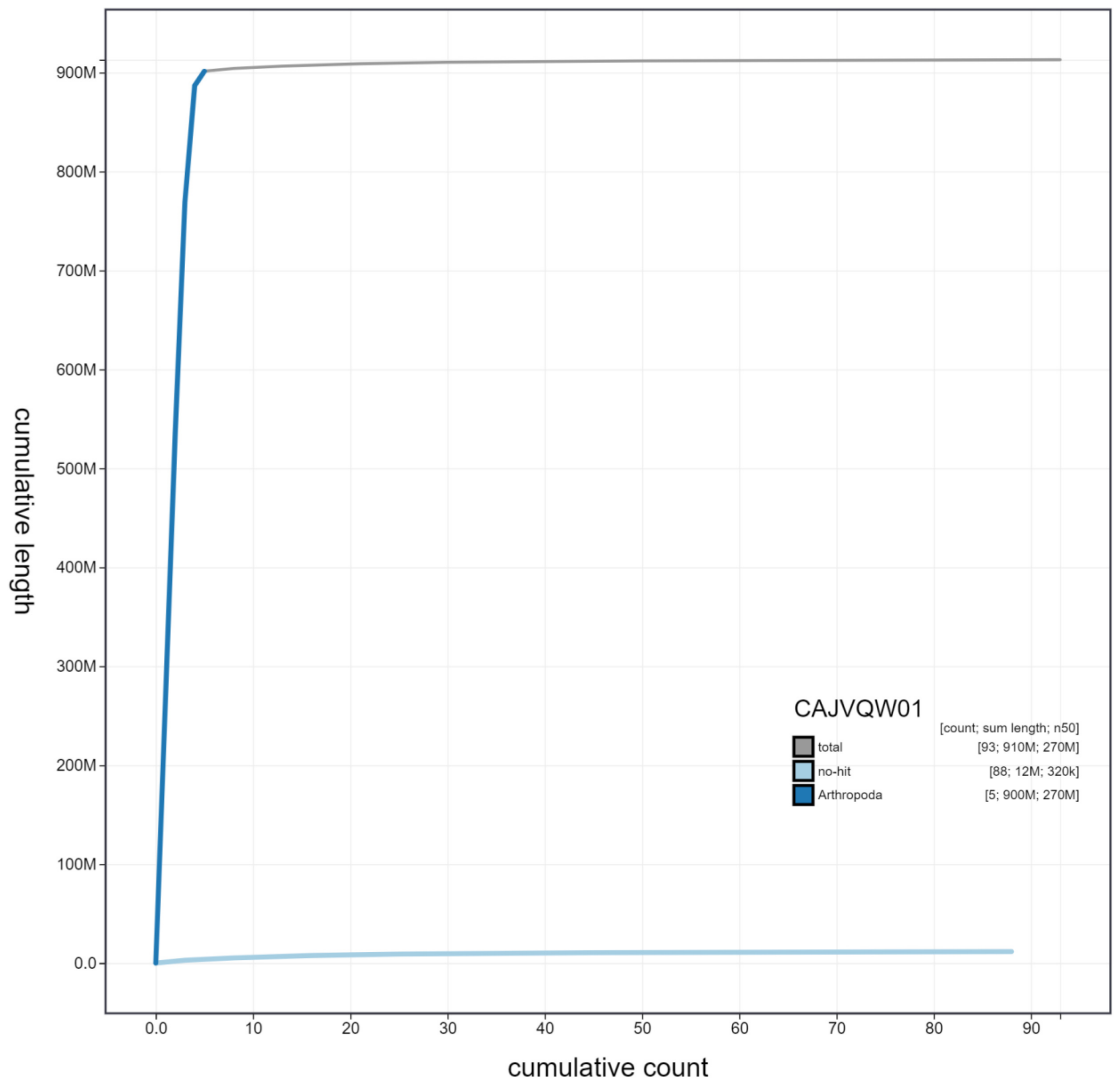
Genome assembly of *Chrysotoxum bicinctum*, idChrBici1.1: cumulative sequence. BlobToolKit cumulative sequence plot. The grey line shows cumulative length for all scaffolds. Coloured lines show cumulative lengths of scaffolds assigned to each phylum using the buscogenes taxrule. An interactive version of this figure is available at https://blobtoolkit.genomehubs.org/view/idChrBici1.1/dataset/CAJVQW01/cumulative.

**Figure 5.**
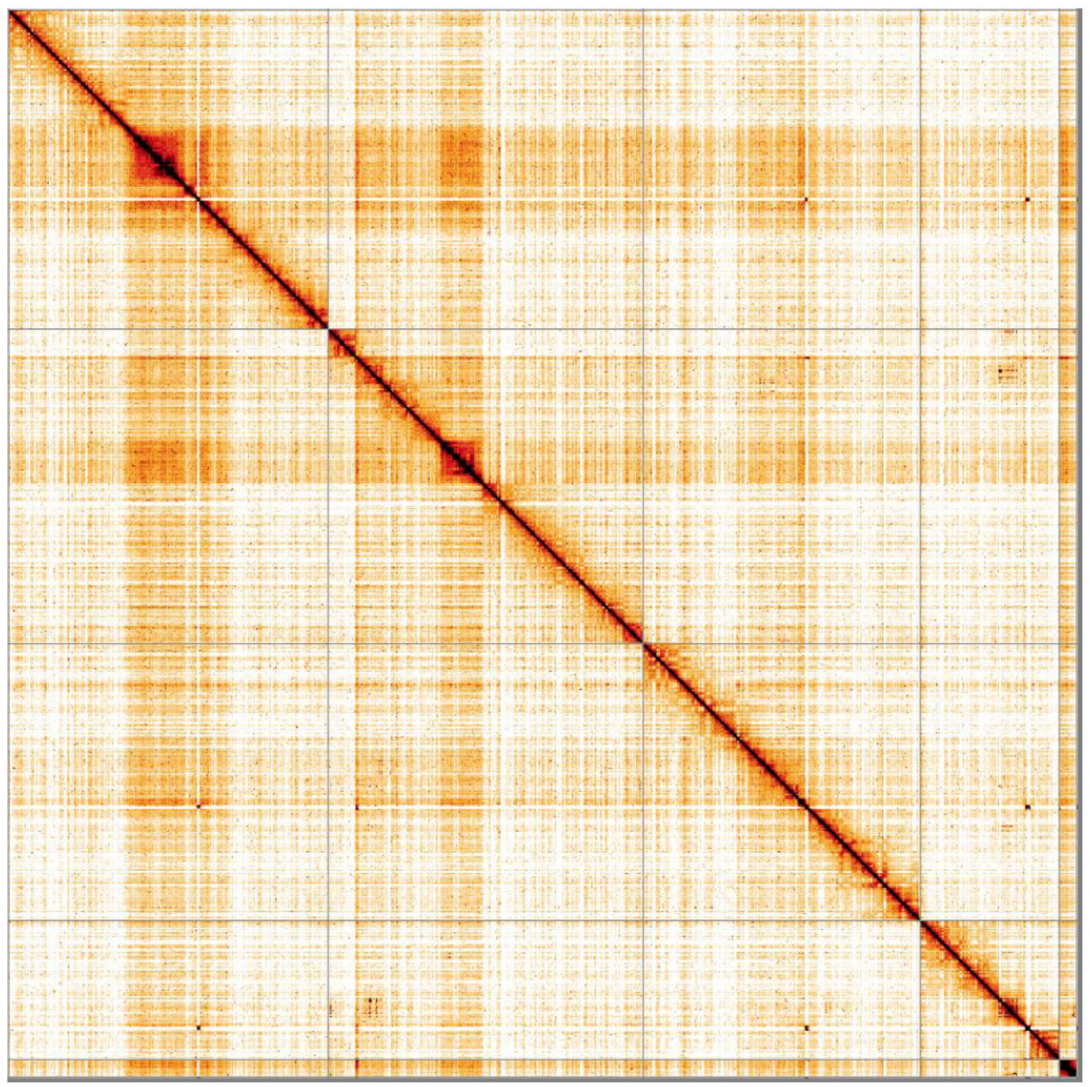
Genome assembly of *Chrysotoxum bicinctum*, idChrBici1.1: Hi-C contact map. Hi-C contact map of the idChrBici1.1 assembly, visualised in HiGlass.

**Table 2.** Chromosomal pseudomolecules in the genome assembly of *Chrysotoxum bicinctum*, idChrBici1.1.

INSDC accession	Chromosome	Size (Mb)	GC%
OU426987.1	1	269.71	34.2
OU426988.1	2	265.79	34.1
OU426989.1	3	233.72	33.9
OU426990.1	4	117.57	34
OU426991.1	X	14.54	33.6
OU426992.1	MT	0.02	17.5
-	Unplaced	11.59	36.8

## Methods

### Sample acquisition and nucleic acid extraction

A female (idChrBici1) *C. bicinctum was* collected from Wytham Great Wood, Oxfordshire, UK (latitude 51.769, longitude -1.33) by Will Hawkes, University of Exeter, who also identified the sample. A second sample of unknown sex (idChrBici2), was collected by Matt Smith from Hartslock Reserve, Oxfordshire, UK (latitude 51.511263, longitude -1.112222). The samples were collected using a net and snap-frozen on dry ice.

DNA was extracted from the whole organism of idChrBici1 at the Wellcome Sanger Institute (WSI) Scientific Operations core from head/thorax tissue using the Qiagen MagAttract HMW DNA kit, according to the manufacturer’s instructions. Following this, further DNA was extracted for a PacBio top-up. Tissue was cryogenically disrupted to a fine powder using a Covaris cryoPREP Automated Dry Pulveriser, receiving multiple impacts. Fragment size analysis of 0.01–0.5 ng of DNA was then performed using an Agilent FemtoPulse. High molecular weight (HMW) DNA was again extracted using the Qiagen MagAttract HMW DNA extraction kit. HMW DNA was sheared into an average fragment size between 12–20 kb in a Megaruptor 3 system with speed setting 30. Sheared DNA was purified by solid-phase reversible immobilisation using AMPure PB beads with a 1.8X ratio of beads to sample to remove the shorter fragments and concentrate the DNA sample. The concentration of the sheared and purified DNA was assessed using a Nanodrop spectrophotometer and Qubit Fluorometer and Qubit dsDNA High Sensitivity Assay kit. Fragment size distribution was evaluated by running the sample on the FemtoPulse system.

RNA was extracted from thorax tissue of idChrBici2 in the Tree of Life Laboratory at the Wellcome Sanger Institute using TRIzol (Invitrogen), according to the manufacturer’s instructions. RNA was then eluted in 50 μl RNAse-free water and its concentration assessed using a Nanodrop spectrophotometer and Qubit Fluorometer using the Qubit RNA Broad-Range (BR) Assay kit. Analysis of the integrity of the RNA was done using Agilent RNA 6000 Pico Kit and Eukaryotic Total RNA assay.

### Sequencing

Pacific Biosciences HiFi circular consensus and 10X Genomics Chromium read cloud sequencing libraries were constructed according to the manufacturers’ instructions. Poly(A) RNA-Seq libraries were constructed using the NEB Ultra II RNA Library Prep kit. Sequencing was performed by the Scientific Operations core at the Wellcome Sanger Institute on Pacific Biosciences SEQUEL II (HiFi), Illumina HiSeq X (10X) and Illumina HiSeq 4000 (RNA-Seq) instruments. Hi-C data were generated from abdomen tissue of idChrBici1, and head and abdomen tissue of idChrBici2 using the Arima Hi-C+ kit and sequenced on HiSeq X (idChrBici1) and Illumina NovaSeq 6000 instruments (idChrBici2).

### Genome assembly

Assembly was carried out with Hifiasm ( Cheng *et al*., 2021); haplotypic duplication was identified and removed with purge_dups ( Guan *et al*., 2020) with the -e flag. One round of polishing was performed by aligning 10X Genomics read data to the assembly with longranger align, calling variants with freebayes ( Garrison & Marth, 2012). The assembly was then scaffolded with Hi-C data ( Rao *et al*., 2014) using SALSA2 ( Ghurye *et al*., 2019). The assembly was checked for contamination and corrected using the gEVAL system ( Chow *et al*., 2016) as described previously ( Howe *et al*., 2021). Manual curation was performed using gEVAL, HiGlass ( Kerpedjiev *et al*., 2018) and Pretext. The mitochondrial genome was assembled using MitoHiFi ( Uliano-Silva *et al*., 2021) and annotated with MitoFinder ( Allio *et al*., 2020). The genome was analysed and BUSCO scores generated within the BlobToolKit environment ( Challis *et al*., 2020). Table 3 contains a list of all software tool versions used, where appropriate.

**Table 3.** Software tools used.

Software tool	Version	Source
Hifiasm	0.15	Cheng *et al*., 2021
purge_dups	1.2.3	Guan *et al*., 2020
SALSA2	2.2	Ghurye *et al*., 2019
longranger align	2.2.2	https://support.10xgenomics.com/genome- exome/software/pipelines/latest/advanced/ other-pipelines
freebayes	1.3.1-17-gaa2ace8	Garrison & Marth, 2012
MitoHiFi	2.0	Uliano-Silva *et al*., 2021
gEVAL	N/A	Chow *et al*., 2016
HiGlass	1.11.6	Kerpedjiev *et al*., 2018
PretextView	0.2.x	https://github.com/wtsi-hpag/PretextView
BlobToolKit	2.6.2	Challis *et al*., 2020

## Data availability

European Nucleotide Archive: Chrysotoxum bicinctum (two-banded wasp hoverfly). Accession number PRJEB45198; https://identifiers.org/ena.embl/PRJEB45198.

The genome sequence is released openly for reuse. The *C. bicinctum* genome sequencing initiative is part of the Darwin Tree of Life (DToL) project. All raw sequence data and the assembly have been deposited in INSDC databases. The genome will be annotated using the RNA-Seq data and presented through the Ensembl pipeline at the European Bioinformatics Institute. Raw data and assembly accession identifiers are reported in Table 1.

## Author information

Members of the University of Oxford and Wytham Woods Genome Acquisition Lab are listed here: https://doi.org/10.5281/zenodo.4789929.

Members of the Natural History Museum Genome Acquisition Lab are listed here: https://doi.org/10.5281/zenodo.4790043.

Members of the Darwin Tree of Life Barcoding collective are listed here: https://doi.org/10.5281/zenodo.4893704.

Members of the Wellcome Sanger Institute Tree of Life programme are listed here: https://doi.org/10.5281/zenodo.5377053.

Members of Wellcome Sanger Institute Scientific Operations: DNA Pipelines collective are listed here: https://doi.org/10.5281/zenodo.4790456.

Members of the Tree of Life Core Informatics collective are listed here: https://doi.org10.5281/zenodo.5013542.

Members of the Darwin Tree of Life Consortium are listed here: https://doi.org/10.5281/zenodo.4783559.
