# Supplementary material for: The genome sequence of the two-banded wasp hoverfly, Chrysotoxum bicinctum (Linnaeus, 1758)
Source: Wellcome Open Res. 2021 Nov 24;6:321. [Version 1] doi: 10.12688/wellcomeopenres.17382.1 (PMC9971694; doi:10.12688/wellcomeopenres.17382.1)
Supplement: Peer review history

# Peer review: Reviewer response for version 1

**DOI:** 10.21956/wellcomeopenres.19219.r54531

**Published:** 2023-02-16

**Authors:** Ann-Marie Waldvogel

**Affiliations:** 1 Institute of Zoology, University of Cologne, Cologne, Germany

Hawkes and colleagues present a genome report for the de novo assembly of a reference genome for *Chrysotoxum bicinctum*, the two-banded wasp hoverfly. Authors provide a good description on the ecology and distribution of the species. Following a state-of-the-art procedure based on Pacbio HiFi, 10X genomics and Hi-C sequencing, the final assembly reaches chromosomal resolution including 4 autosomes and one sex chromosome. The assembly is partially phased.

 Despite the availability of RNA-Seq data, the nuclear assembly has not been structurally annotated. This step might be valuable to add now or in close future. 

 Data is available at the European Nucleotide Archive.

Are sufficient details of methods and materials provided to allow replication by others?

Yes

Is the rationale for creating the dataset(s) clearly described?

Yes

Are the datasets clearly presented in a useable and accessible format?

Yes

Are the protocols appropriate and is the work technically sound?

Yes

Reviewer Expertise:

ecological genomics, population genomics

I confirm that I have read this submission and believe that I have an appropriate level of expertise to confirm that it is of an acceptable scientific standard.

# Peer review: Reviewer response for version 1

**DOI:** 10.21956/wellcomeopenres.19219.r50745

**Published:** 2022-06-13

**Authors:** Shu-Jun Wei, Li-Jun Cao

**Affiliations:** 1 Institute of Plant Protection, Beijing Academy of Agriculture and Forestry Sciences, Beijing, China

This data note reports the genome of the two-banded wasp hoverfly *Chrysotoxum* *bicinctum*. The hoverflies are very interesting species of insects. Genomes of hoverflies are usually large and difficult to assemble. I am happy to see the publication of this hoverfly genome. The genome was assembled using long reads, linked reads, and Hi-C technologies. Data were well analyzed using available methods and software. The results will provide invaluable resources for hoverflies' studies and references for other relative species' genome assembly. I have some minor comments.

1. The genome was assembled into five pseudochromosomes with the X sex chromosome. What is the karyotype of this species or other relative species? How did the authors make sure the identified X chromosome is correct? There is a lack of analysis of the X chromosome to confirm the identification.
2. Sampling information was repeated in “Genome sequence report” and “Methods” sections. Remove one of the duplicated contents.
3. I noticed that many figures of this manuscript were generated by BlobToolKit Pipeline and used directly. However, there are many format problems in these figures. I suggest the author revise these figures carefully. Some typical issues: Figure 3, change “gc” to “GC content”, sum to “Sum”, “total” to “Total”, “no-hit” to “No-hit”. The same problems were found in other figures where the first letters were not capitalized for the first word. Figure 5, length in Mb should be marked in the axes.

Are sufficient details of methods and materials provided to allow replication by others?

Yes

Is the rationale for creating the dataset(s) clearly described?

Yes

Are the datasets clearly presented in a useable and accessible format?

Yes

Are the protocols appropriate and is the work technically sound?

Yes

Reviewer Expertise:

I work on insect genomics, population genetics, and pest control research. I used population genomics approaches to trace insects' origin, invasion routes, long-distance migration, and local adaptation to pesticide and environmental stresses.

We confirm that we have read this submission and believe that we have an appropriate level of expertise to confirm that it is of an acceptable scientific standard.
